# Exercise modulates brain pulsatility: insights from q-aMRI and MRI-based flow methods

**DOI:** 10.1098/rsfs.2024.0043

**Published:** 2025-04-04

**Authors:** Jethro Stephan Wright, Edward Clarkson, Haribalan Kumar, Itamar Terem, Alireza Sharifzadeh-Kermani, Josh McGeown, Ed Maunder, Paul Condron, Gonzalo Maso Talou, David Dubowitz, Miriam Scadeng, Sarah-Jane Guild, Vickie Shim, Samantha J. Holdsworth, Eryn Kwon

**Affiliations:** ^1^ Matai Medical Research Institute, Tairāwhiti-Gisborne, New Zealand; ^2^ Auckland Bioengineering Institute, University of Auckland, Auckland, New Zealand; ^3^ General Electric Healthcare, Tairāwhiti-Gisborne, New Zealand; ^4^ Department of Electrical Engineering, Stanford University, Stanford, CA, USA; ^5^ Faculty of Medical and Health Sciences, University of Auckland, Auckland, New Zealand; ^6^ Sports Performance Research Institute, Auckland University of Technology, Auckland, New Zealand; ^7^ University of Auckland, Auckland, New Zealand; ^8^ Auckland Bioengineering Institute, University of Auckland, Auckland, New Zealand; ^9^ Centre for Advanced MRI (CAMRI), University of Auckland, Auckland, New Zealand; ^10^ Department of Anatomy and Medical Imaging, Faculty of Medical and Health Sciences, University of Auckland, Auckland, New Zealand

**Keywords:** brain motion, brain pulsatility, cerebral blood flow, cerebrospinal fluid, exercise, amplified magnetic resonance imaging

## Abstract

This study investigates intracranial dynamics following the Monro–Kellie doctrine, depicting how brain pulsatility, cerebrospinal fluid (CSF) flow and cerebral blood flow (CBF) interact under resting and exercise conditions. Using quantitative amplified magnetic resonance imaging (q-aMRI) alongside traditional MRI flow metrics, we measured and analysed blood flow, CSF dynamics and brain displacement in a cohort of healthy adults both at rest and during low-intensity handgrip exercise. Exercise was found to reduce pulsatility in CBF while increasing CSF flow and eliminating CSF regurgitation, highlighting a shift towards more sustained forward flow patterns (from cranial to spinal compartments). Displacement analysis using q-aMRI revealed a consistent trend of reduced whole brain motion during exercise, though as the sample of data that met quality control was low (*n* = 5), this was not a significant result. There was an observable decrease in the motion of third and fourth ventricles, linking ventricular displacement to CSF flow alterations. These findings suggest that exercise may not only affect the rate and directionality of CSF flow but also modulate brain tissue motion, supporting cerebral homeostasis. This study offers insights into how the brain adapts dynamically under varying conditions, with implications for understanding intracranial pressure regulation in humans and diagnostic contexts.

## 1. Introduction

The cranium houses three components that have a homeostatic relationship: brain tissue, cerebrospinal fluid (CSF) and blood [1]. Changes to any one of these components will alter intracranial pressure or initiate a reciprocal change that adjusts the other components to maintain constant pressure. This relationship is known as the Monro–Kellie doctrine [2,3]. This doctrine considers changes in volume at a steady-state equilibrium, where the net volume exchange of fluids into and out of the brain is zero, making variations in the total flow of these fluids negligible [2,3]. However, there are transient temporal changes in the partial volume of each intracranial compartment, mainly driven by cardiac input. The improved temporal resolution of fluid flow techniques and other means to visualize brain motion allow us to consider a more comprehensive model for emerging research fields such as brain pulsatility.

Based on the Monro–Kellie doctrine where constituents are homeostatically balanced in terms of total volume, it can be inferred that related dynamics (such as cerebral blood flow (CBF), CSF flow and brain motion) are also linked. We operationally define these factors as *intracranial dynamics*, and this paper intends to visualize these.

### 1.1. Intracranial constituents and dynamics

Within the intracranial space, the volume of blood and CSF is comparable. However, CBF entering and subsequently leaving the brain is of much larger magnitude than CSF being produced and reabsorbed (2000 : 1). Although this study does not comprehensively investigate the CBF and CSF systems, the flow differences should be noted when investigating intracranial dynamics and relative flow changes. For example, relatively small percentage changes in blood flow may be correlated to much larger percentage changes in CSF flow.

Net CBF and CSF flow trade-off can be explained by the traditional Monro–Kellie doctrine but it does not predict the dynamic behaviour of CBF and CSF flow. These dynamic changes in flow along with brain tissue motion are the subject of investigation in this paper.

Parenchymal brain tissue is a somewhat compressible material with elastic properties [4] and increases in intracranial pressure will result in compression of the brain [5]. Similarly, it has long been established that the brain moves over a cardiac cycle [6–9]. Further studies have also linked parenchymal motion to CBF and CSF flow [10–14]. However, the relationship between exercise-related changes in parenchymal motion and CBF/CSF flow has yet to have been explored.

**Figure 1.**
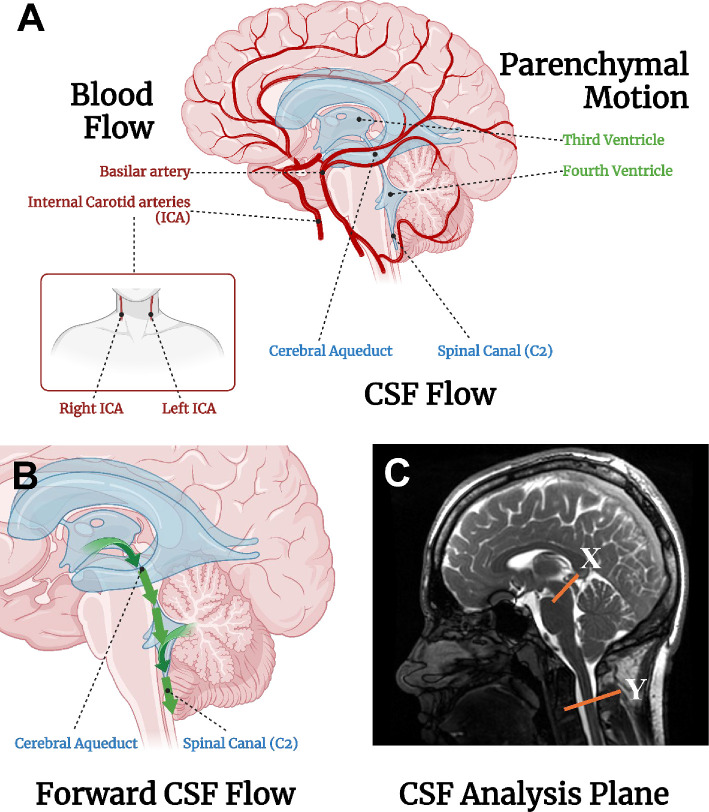
(A) Regions of CBF, CSF flow and parenchymal motion that were measured and subsequently analysed in this study. Regions were chosen due to their close proximity and physiological relationships. CBF of the basilar artery is correlated to CSF flow in the cerebral aqueduct and displacement of the third ventricle. CBF of the ICA is correlated to CSF flow in the spinal canal (C2) and displacement of the fourth ventricle. (B) The forward flow of CSF: the flow of CSF in the direction of the green arrows is considered positive flow. Flow opposite this is considered negative flow, or regurgitation. (C) The two-dimensional phase contrast planes of CSF flow recording. X = cerebral aqueduct, Y = C2 of the spinal canal. Created in BioRender. Wright, J. (2025) BioRender.com/q03r951 (A) and BioRender.com/m70e468 (B,C).

Based on the established link between CBF, CSF flow and brain motion, this study identifies a few key regions where these dynamics may be observed (figure 1). These regions were partially based on a similar study looking at CBF and CSF flow in exercise [15]. Blood vessels chosen were also based on being a large enough diameter to accurately measure with a short blood flow MRI sequence. The internal carotid arteries (ICAs) run alongside the spinal cord into the intracranial space, which may be related to the parenchymal motion of the fourth ventricle and CSF flow at C2 of the spine. The basilar artery bifurcates into two smaller arteries that run perpendicular to the cerebral aqueduct, so may be related to the CSF flow of the aqueduct and the motion of the third ventricle.

### 1.2. Amplified MRI and quantitative amplified MRI

Amplified MRI (aMRI) allows the visualization and quantification of subtle physiological brain motions, providing unique insights into brain pulsatility patterns [10–14]. This technique enhances sensitivity to subvoxel displacements, detecting and analysing fine details of brain dynamics by selectively amplifying motion associated with specific frequencies. By tuning to cardiac harmonics, aMRI can visualize minute brain displacements linked to the cardiac cycle, highlighting the relationship between brain motion, CSF dynamics and blood flow. aMRI-based measurements of brain pulsatility align well with established methods, providing complementary data that enhances the understanding of brain dynamics [10–14].

Initially, aMRI provided qualitative insights into brain motion patterns, averaged across the heartbeat cycle [10,11]. Recently, quantitative aMRI (q-aMRI) was developed, allowing for direct quantification of these subvoxel motions in three-dimensional space [14]. This enables observation of displacement patterns in the whole brain and in specific regions of interest (ROIs). Key ROIs include the third and fourth ventricles—crucial channels for CSF flow into the cerebral aqueduct and spinal canal—and the brainstem, known for pronounced movement during the cardiac cycle [6–14].

q-aMRI extends aMRI by creating detailed maps of brain displacement, describing both the extent and direction of motion across brain regions [14]. These maps enable the extraction of displacement data from specific ROIs, such as central CSF-rich areas. Currently, no studies have directly compared brain pulsation with CSF and blood flow measurements.

The brain operates as a complex system that maintains a delicate balance of volumes and dynamics [14,16–18]. This balance is sensitive to the regulated inflow, outflow and total volumes of CSF and blood. Variations in these factors can significantly impact brain function and tissue motion and investigating these flow dynamics alongside brain tissue displacement allows for deeper insights into how these forces interact. Such investigations may reveal new aspects of the brain’s biomechanical environment, advancing our understanding of its dynamic balance and systemic physiology.

To improve understanding of intracranial dynamics, important parameters such as CBF, CSF flow and brain motion in both resting and exercise conditions have been investigated. The key hypotheses were as follows.

(1) CBF and CSF flow dynamics are affected by exercise (as seen in a similar study by Tarumi *et al*. [15] and found in other related literature [19–29]). Likewise, changes will also be observed in parenchyma motion as measured by q-aMRI displacement.
(2) There are observable similarities between the patterns of CBF and/or CSF flow and q-aMRI displacement, suggesting a relationship between these intracranial dynamics.

## 2. Methods

### 2.1. Participants

With approval from the New Zealand Health and Disability Ethics Committee (reference number: 20/CEN/107) and informed consent, 17 participants were recruited for this study. Participants were healthy young adults (10 female and 7 male), with an age range of 18−23 years. Exclusion criteria for study participation were any underlying health conditions, and standard MRI contraindications (e.g. implants). All 17 participants were included in the CBF analysis and CSF flow analysis.

Since q-aMRI is very susceptible to motion, this study had strict inclusion/exclusion criteria. As part of quality control, participants were excluded if the raw data contained significant temporal flickering, or if motion artefacts such as movement of the skull were present in the aMRI video outputs. Scans from five participants were deemed suitable for q-aMRI processing; therefore only five participants were included in the comparison between q-aMRI and fluid flow patterns.

### 2.2. Exercise task

**Figure 2.**
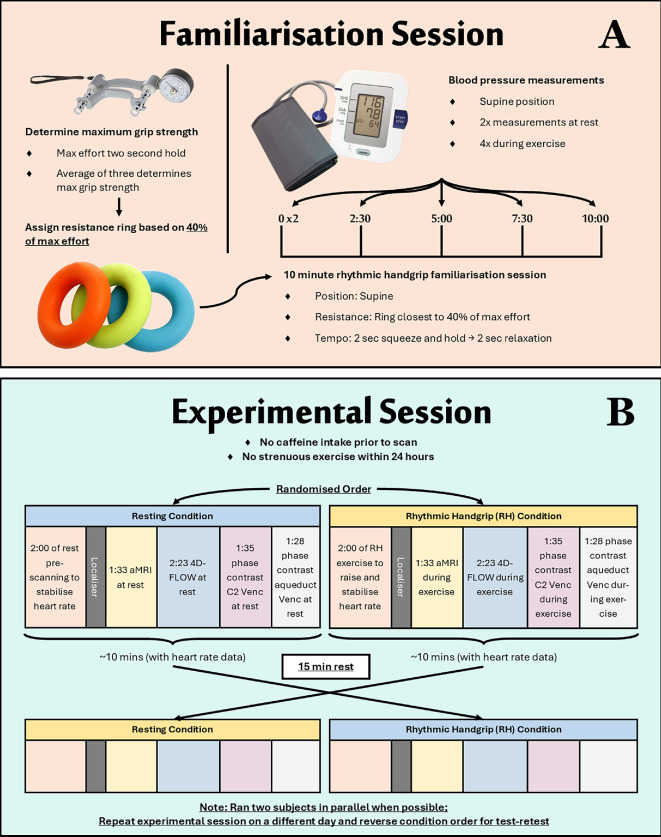
Outline of the (A) familiarization session and (B) experimental session participants performed. Participants who were assigned the resting condition followed with the RH condition after rest (blue to blue), while participants who were assigned the RH condition first followed with rest (yellow to yellow).

After trialling multiple exercise tasks, a rhythmic handgrip exercise based on Tarumi *et al*. [15] was selected as it reduced the amount of motion artefacts [15]. Participants performed a familiarization session prior to the experimental data collection where maximum grip strength was determined for each participant by using a hand grip strength dynamometer figure 2A. Participants were assigned a squeezable resistance ring with a compression force closest to 40% of their maximum strength as this was found to be sustainable for the 10 min session. The handgrip exercise was performed with the dominant hand. Blood pressure was measured before and after the MRI. Heart rate was monitored via peripheral cardiac gating.

On the day of participation, participants were rested and had not ingested caffeine prior to the scan. Participants were randomly assigned to either the resting or handgrip condition during their first MRI scan. They then had a 15 min rest outside the scanner, before completing the second condition in the MRI scanner. During the test of methods phase, participants had repeat experimental sessions on a different day with the reverse condition to ensure test–retest reliability (figure 2B).

### 2.3. MRI acquisition

The experimental sessions were performed in a 3 T MRI machine (SIGNA Premier; General Electric Healthcare, Milwaukee, WI) and a 48-channel AIR™ coil. Two minutes were allocated to heart rate stabilization in both resting and exercising conditions before initiation of the scanning procedure. Selected MRI scans collected during the acquisition which are relevant to the current study are shown in table 1 and include T1-weighted structural imaging, q-aMRI (whole brain), blood flow via four-dimensional phase contrast MRI (collection window spanning from neck to superior regions of the brain), and two-dimensional CSF flow at the C2 spine and cerebral aqueduct based on the acquisition described in Tarumi *et al*. [15]. Flow and displacement measurements start and end at the *R* peaks (*R–R* wave) of ECG recordings and are averaged across multiple heartbeats.

**Table 1.** Imaging sequences and parameters used in the experimental session during scanning with 3 T MRI.

imaging sequence and region captured	pulse sequence (*generic name*)	FOV (mm)	resolution (mm)	TR/TE/flip angle	additional imaging-specific parameters	time (min)
3D T1 structural image (whole brain)	BRAVO (*3D IR-prep fSPGR*)	240	1.0 × 1.0 × 1.0 interpolated to 0.5 × 0.5 × 0.5	7 ms/2.8 ms/8°	TI = 600 ms parallel imaging factor (*R*) = 2	1:44
3D q-aMRI (whole brain) (for capturing brain motion)	3D CINE-FIESTA (*balanced SSFP*)	256	1.0 × 1.0 × 1.0	2.8 ms/1 ms/25°	peripheral cardiac gating (PG) with 20 cardiac phases hyperkat = 8	1:33
4D blood flow (proximal to the brainstem and circle of Willis)	4D FLOW (*cine 3D phase-contrast*)	220	1.2 × 1.2 × 1.4	4.1 ms/2.4 ms/8°	PG with 16 (reconstructed to 20) cardiac phases NEX = 4 Venc = 80 cm s^−1^ flow direction: S–I, A–P, R–L hyperkat = 8	2:23
CSF flow at the C2 level (single axial slice)	vasc PC (*2D phase-contrast*) axial slice	180 × 144 with 0.8 phase FOV	0.7 × 0.7 × 4	9.8 ms/5.5 ms/20°	PG with 30 cardiac phases, 2 views per segment venc = 9 cm s^−1^ flow direction: S–I *R* = 2	1:35
CSF flow at aqueduct level (single axial slice)	vasc PC (*2D phase-contrast*) axial slice	180 × 162 with 0.8 phase FOV	0.9 × 0.9 × 4	9.0 ms/5.4 ms/20°	PG with 30 cardiac phases, 2 views per segment venc = 9 cm s^−1^ flow direction: S–I *R* = 2	1:28

### 2.4. Cerebral blood flow analysis

**Figure 3.**
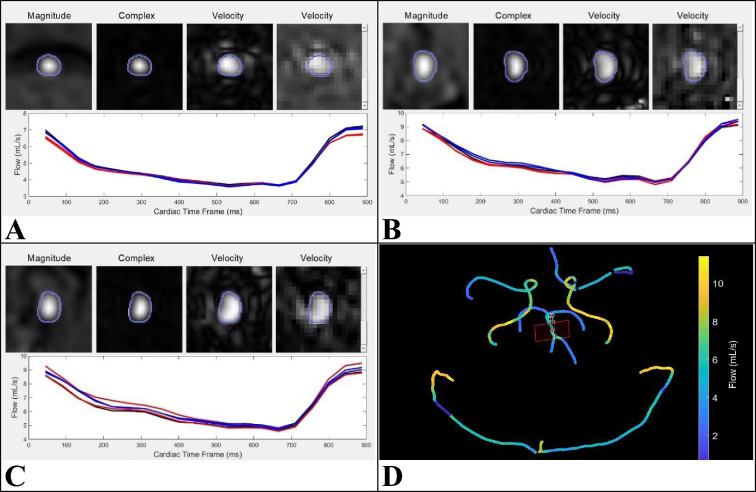
QVT analysis window of the (A) basilar artery, (B) right ICA and (C) left ICA. (A–C) This displays the flow profile over a heart-beat where the first three panels (above line graph) are used for overall quality control, while the last velocity panel allows for frame-by-frame quality control. The black line shows the flow data from the cross-section selected for analysis, whilst the blue and red lines show the two previous and subsequent cross-sections respectively (ensuring accuracy). (D) Displaying the QVT vessel selection screen, where the selected cross-section is outlined by the red square. Flow rate (ml s^−1^) is displayed as a colour gradient (scale to the right of the image). doi.org/10.5281/zenodo.14715242.

Four-dimensional flow measures pulsatile blood flow in cerebral vasculature in a three-dimensional volume, over a representative cardiac cycle. To extract regional blood flow from the vasculature, a quantitative velocity tool (QVT) was used (figure 3) [30,31]. QVT provided a more reliable estimate of blood flow, by reducing the human error of manual ROI placements that other clinical software requires.

CBF values were obtained by performing QVT analysis on the four-dimensional flow data which were captured across 20 cardiac phases. Analysis was performed for three ROIs (basilar artery, left ICA and right ICA) in all participants, for both the resting and exercise conditions. These ROIs were chosen as they are the major sources of blood flow to the intracranial space. Additionally, the ROIs are located in close proximity to the CSF ROI (figure 1), and there is a high flow in these vessels. The basilar artery leads to the posterior cerebral artery which runs next to the cerebral aqueduct in the mid-sagittal section of the brain, while the two ICAs run up alongside the spinal column and are the main source of blood entering the intracranial space (figure 1). The recordings of both ICAs were summed together to get the total blood flow value.

### 2.5. Cerebrospinal fluid flow analysis

An outline was manually traced on a single slice around the CSF compartments of the aqueduct and the subarachnoid space at the level of C2. This was performed by a single MRI technologist with over 20 years of experience. A static tissue mask correction was applied to delineate the CSF compartments and aid in tracing the compartment boundary. Background correction and anti-aliasing correction included within CVI42 software were also applied to reduce inherent errors during acquisition such as eddy currents, gradient field effects and phase offset, as well as reducing aliasing artefacts that can decrease the accuracy of flow parameters. The two ROI masks were separately applied to the MRI image and measurements of CSF flow and velocity were calculated across all cardiac frames.

### 2.6. Quantitative amplified MRI for brain displacement analysis

Each three-dimensional cine dataset (acquired using three-dimensional balanced SSFP) underwent motion extraction with the q-aMRI algorithm described in [14]. The resulting displacement maps contain vectors describing motion for each cardiac frame. The volume at the first cardiac phase also underwent automatic brain segmentation with FreeSurfer’s multi-contrast segmentation tool [32]. Displacement within the third ventricle, fourth ventricle and CSF regions defined in the Desikan Killiani parcellation scheme [33] were extracted, just as in the study by [14]. Lastly, brain extraction with the multi-contrast tool HD-BET was performed on the images to remove non-brain regions [34]. Cardiac frames with the greatest total positive displacement within the brain mask in the superior–inferior direction were calculated. The median displacement in the superior–inferior direction normalized to the 99th percentile maximum displacement observed was compared between exercise and rest with the non-parametric Wilcox test. The median was selected as a measure of centrality due to the skewed distribution of the displacement data.

### 2.7. Visualization of flow patterns and statistical analysis

Four-dimensional flow and q-aMRI were recorded over 20 cardiac phases while CSF flow was recorded over 30 phases. Interpolated downsampling of CSF flow was performed to produce 20 cardiac frames for comparability. These data were analysed and plotted for visualization.

Area under the curve (AUC) for CBF and CSF flow was calculated using Simpson’s rule (accurate for nonlinear data) in Python [35]. AUC is used to measure cumulative flow, where increased AUC means greater flow over a cardiac cycle:


$$AUC\approx \frac{\Delta x}{3}(y_{1}+4y_{2}+2y_{3}+4y_{4}+\cdots +y_{n}).$$

The pulsatility index (PI) is defined as the difference between the peak systolic and minimum diastolic flow, divided by the mean overall flow [21]. PI is particularly adept at analysing peak-to-trough variation and can provide insight into more subtle fluctuation pattern changes. Additionally, PI can inform resistance and cardiac function, although this paper does not fully explore these inferences. The PI calculation was performed on the CBF in the basilar artery and ICA. The PI was not applied to the CSF data.

Whole brain maximum displacement calculations were performed using q-aMRI. Larger maximum displacement indicates greater movement of the brain in a cardiac cycle. Subsequently, two ROIs on the third and fourth ventricles were segmented, and the q-aMRI displacement was calculated in the anterior–posterior (A–P), superior–inferior (S–I), and left–right (L–R) directions.

Bivariate correlation analyses using Spearman’s rho were performed in R-Studio for comparing intracranial dynamics.

## 3. Results

The average heart rate for rest was 69.4 bpm (14.3 std), and exercise was 80.4 (15.3 std). The heart rate increased by an average of 16.6% (11.5% std) from rest to exercise states.

### 3.1. Basilar artery cerebral blood flow

**Figure 4.**
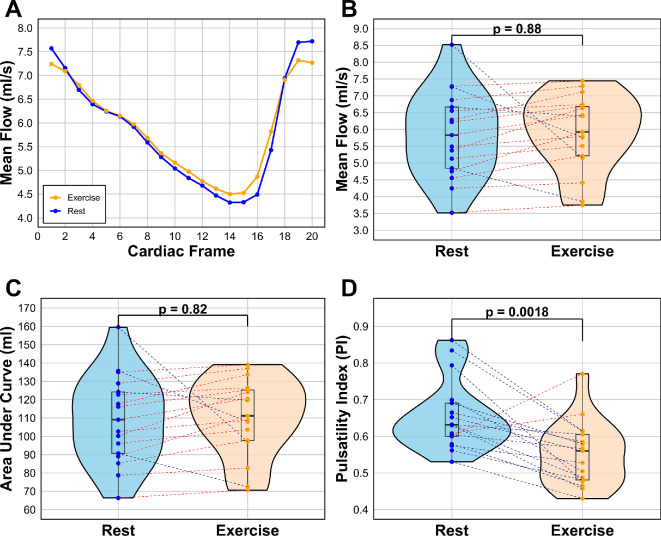
Blood flow of the basilar artery for rest and exercise, showing (A) line graph of the average flow pattern of all participants, tracking the flow across cardiac frames 1−20 of the cardiac cycle (*R–R* peak), (B) mean flow for each participant in ml s^−1^ by averaging the 20 cardiac frames, (C) AUC comparison for each participant, and (D) the difference in PI between rest and exercise. Blue = rest, orange = exercise.

Figure 4 illustrates no observable difference in the overall flow pattern (figure 4A) in the basilar artery between rest and exercise, based on the average of all participants across the cardiac cycle (*R*–*R* peak). However, slight variations in peak and trough values are noted. Similarly, there is no significant difference in mean blood flow (figure 4B) or the AUC measurements (figure 4C) between rest and exercise. Notably, a significant reduction in pulsatility is observed during exercise (figure 4D), with a mean PI of 0.656 (0.0919 std) at rest compared to 0.552 (0.0851 std) during exercise (*p* = 0.0018).

### 3.2. Internal carotid artery cerebral blood flow

**Figure 5.**
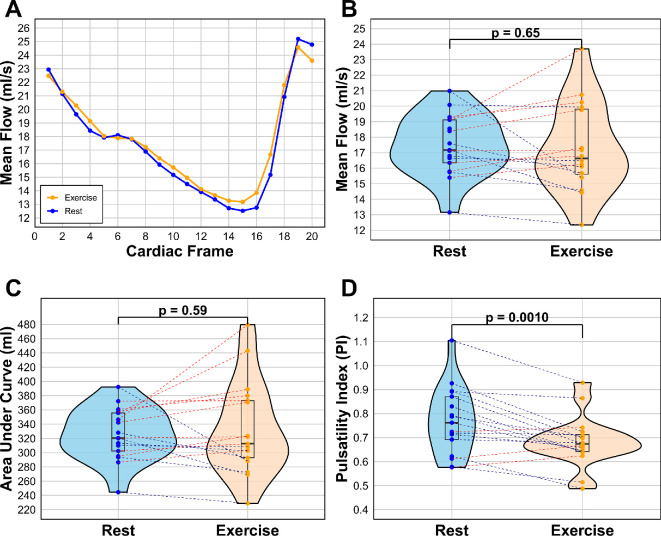
Blood flow of the internal carotid artery for rest and exercise, showing (A) line graph of the average flow pattern of all participants, tracking the flow across cardiac frames 1−20 of the cardiac cycle (*R–R* peak), (B) mean flow for each participant in ml s^−1^ by averaging the 20 cardiac frames, (C) AUC comparison for each participant and (D) the difference in PI between rest and exercise. Blue = rest, orange = exercise.

From figure 5, there is no observable difference in flow pattern between rest and exercise (figure 5A), except the peak flow is higher and trough is lower in rest, thereby the overall range of flow is greater. There is no significant difference in mean flow (figure 5B). There was no significant difference in the AUC measurement (figure 5C), indicating a similar cumulative flow. There is decreased pulsatility in exercise (figure 5D): the mean PI for rest is 0.771 (0.136 std), while the mean for exercise is 0.683 (0.102 std), which suggests less variable flow (*p* = 0.0010).

### 3.3. Aqueduct cerebrospinal fluid flow

**Figure 6.**
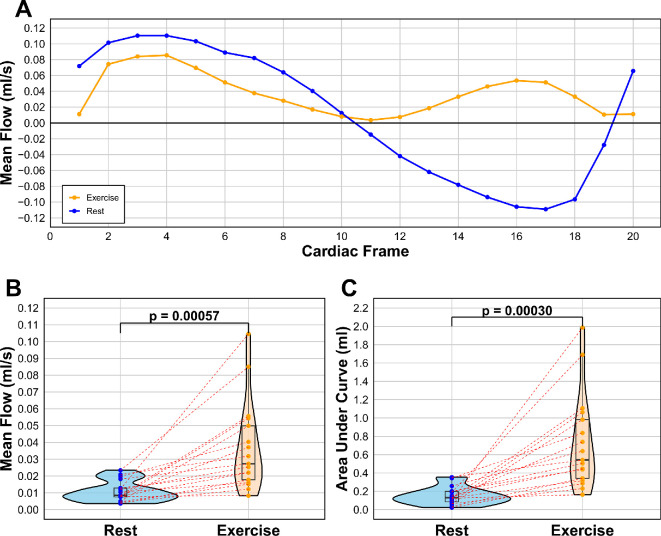
CSF flow of the cerebral aqueduct for rest and exercise, showing (A) line graph of the average flow pattern of all participants, tracking the flow across cardiac frames 1−20 of the cardiac cycle (*R–R* peak), (B) mean flow for each participant in ml s^−1^ by averaging the 20 cardiac frames, and (C) AUC comparison for each participant. Blue = rest, orange = exercise.

Figure 6 shows results from CSF flow measured at the cerebral aqueduct, comparing rest (blue) and exercise (orange) for all 17 participants. Flow appears to follow a different pattern between rest and exercise (figure 6A). When analysing features, rest has one peak and a trough which dips far into negative flow which indicates a large amount of regurgitation. Meanwhile, exercise has two peaks, and flow does not dip below zero meaning there is no regurgitation during exercise. Furthermore, there is a significant difference in mean flow (figure 6B), with an average velocity of 0.0110 ml s^−1^ (0.00596 std) in rest and 0.0368 ml s^−1^ (0.0256 std) in exercise. Regarding AUC (figure 6C), there is a significant difference with an AUC measure of 0.147 (0.0969 std) for rest, and 0.730 (0.511 std) for exercise, indicating a large increase in cumulative net flow during exercise.

### 3.4. C2 spine cerebrospinal fluid flow

**Figure 7.**
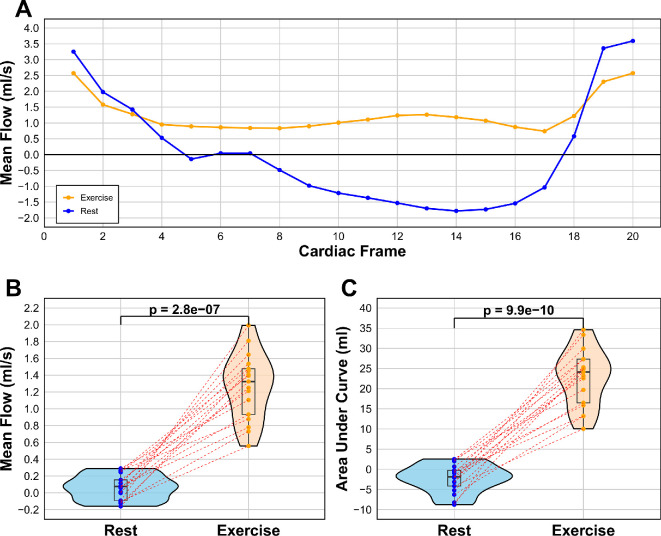
CSF flow of the C2 spine for rest and exercise, showing (A) line graph of the average flow pattern of all participants, tracking the flow across cardiac frames 1−20 of the cardiac cycle (*R–R* peak), (B) mean flow for each participant in ml s^−1^ by averaging the 20 cardiac frames, and (C) AUC comparison for each participant. Blue = rest, orange = exercise.

Figure 7 shows results from CSF flow measured at the C2 spinal level comparing rest and exercise states. There is a stark difference in flow profile (figure 7A) and mean flow (figure 7B) between rest (0.064 ml s^−1^, 0.145 std) and exercise (1.264 ml s^−1^, 0.382 std). Furthermore, when analysing the flow profile, the exercise state showed two peaks, and the trough for rest showed regurgitation of CSF flow. AUC curves confirmed the difference (figure 7C), indicating a significantly changed cumulative net flow, where rest had a net negative AUC (−2.272, 3.44 std), while exercise had a much greater net positive value (22.606, 7.10 std), meaning rest saw more regurgitation than forward flow. There was also large intersubject variability in AUC exercise ranging from 10 to 35.

### 3.5. Quantitative amplified MRI results

**Figure 8.**
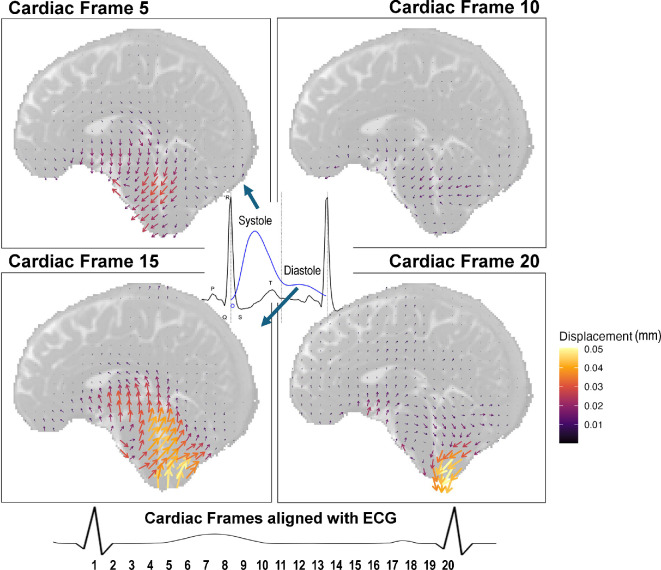
The displacement map for a single subject in the exercise condition is visualized as a vector field. In-plane superior–inferior and anterior–posterior movement is amplified 200 times to illustrate cohesive motion patterns. The motion map has also been down-sampled by a factor of 8. Arrows are coloured according to their total length. The predominant motion at cardiac frame 5 can be seen reversing at cardiac frame 15. Below is a generalized alignment of the 20 cardiac frames of aMRI with an ECG. Note that the cardiac frames start and finish at the *R–R* peaks.

Figure 8 illustrates typical displacement patterns within a q-aMRI map. The pulsatile nature of the motion can be seen from the shift in motion direction at cardiac frame 5 and at cardiac frame 15 (out of 20 total). Significant motion can be seen in the midbrain regions, where motion follows a caudal and rostral trajectory. Cardiac frames 5 and 15 show systolic and diastolic peaks, respectively, while 10 and 20 occur at the transition between systole and diastole.

### 3.6. Whole brain and ventricular displacement

**Figure 9.**
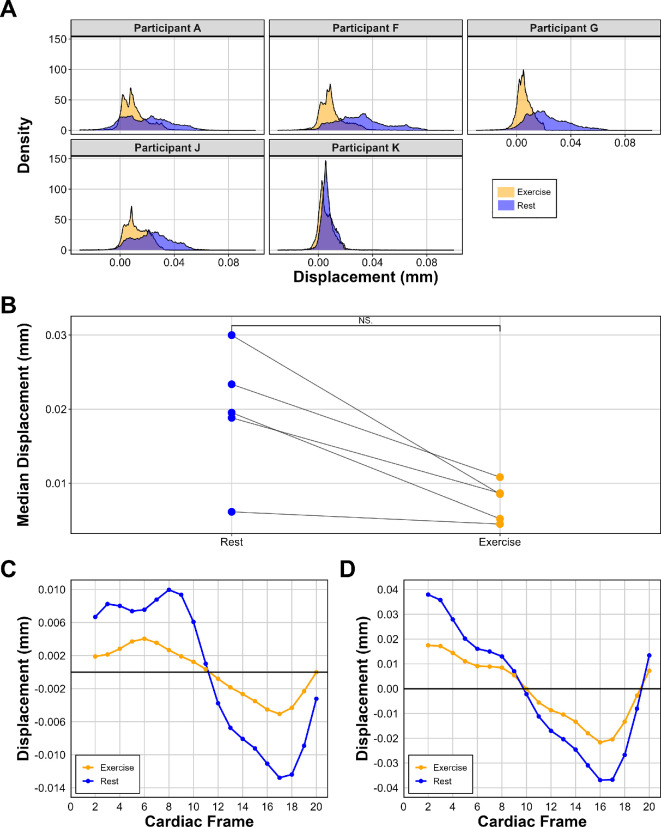
Displacement data from participants who met the inclusion criteria (*n* = 5). (A) Total brain displacement at the cardiac frame with the maximum average displacement. (B) Decreases in median whole brain displacement from rest to exercise. This decrease was not significant (*p* = 0.063). Displacement is calculated in mm. Blue = rest, orange = exercise. (C) The average AP displacement of the third ventricle of all participants. (D) The average SI displacement of the fourth ventricle of all participants. Note: direction of movement for (C,D) was chosen based on the plane of maximum movement for that region.

Figure 9A,B shows the overall displacement of all brain tissue voxels (across all MRI slices as a three-dimensional volume) at the time of maximum average displacement. The distribution of displacement in the exercise condition shows a negative shift (figure 9A), and the median whole brain displacement at the time of maximum displacement decreased in the exercise condition (figure 9B). This decrease was not significant when tested with a Wilcoxon signed-rank test (*p* = 0.063). Although not significant, displacement decreased for all participants in the exercise condition. A larger sample is required to validate this finding.

Figure 9C,D shows the displacement of the third and fourth ventricles, respectively. Only the plane of major motion is displayed (anterior to posterior/AP for the third ventricle and superior to inferior/SI for the fourth ventricle). Further, these planes of motion align with the direction of CSF drainage into the cerebral aqueduct for the third ventricle and the C-spine for the fourth ventricle. Overall, for both the third and fourth ventricles, the exercise state shows reduced motion compared to the resting state, as reflected by a much greater amplitude of displacement in rest (higher peaks and lower troughs). However, the patterns of displacement are similar.

### 3.7. Bivariate analyses with Spearman’s rho

To perform further analyses of intracranial dynamics, data were split into two major ROIs based on their proximal association—third ventricle region and fourth ventricle region. For the first ROI, dynamics measured include displacement of the third ventricle, CBF of the basilar artery and CSF flow of the cerebral aqueduct. For the second ROI, displacement of the fourth ventricle, ICA CBF and C2 spinal CSF flow were measured. Bivariate analyses were performed between all three variables for each ROI to determine the correlation between these dynamics.

**Figure 10.**
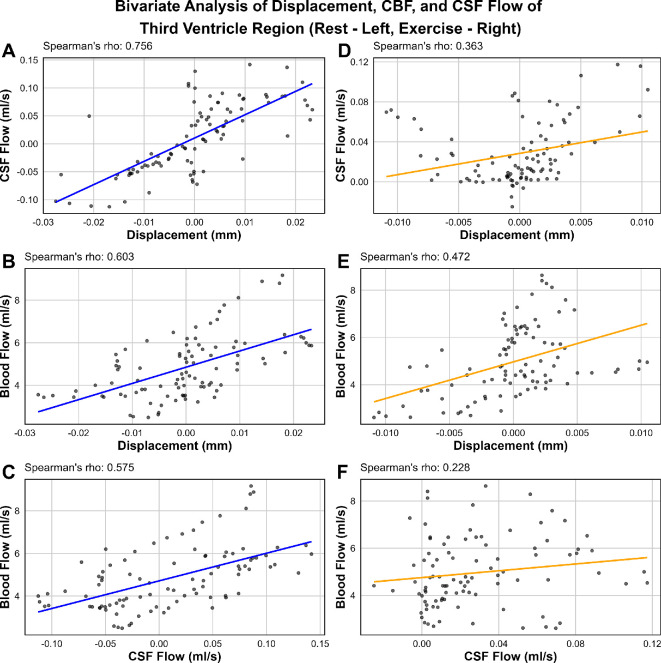
Scatter plots and bivariate correlation analyses using Spearman’s rho of displacement of the first ROI: the third ventricle, CBF of the basilar artery and CSF flow of the cerebral aqueduct in rest (A–C) and exercise (D–F). Note that the data points from all five participants that met q-aMRI criteria for displacement were incorporated into these tests.

Figure 10 displays the bivariate analyses performed on the first ROI. During rest, CSF flow and displacement have a strong correlation (Spearman’s rho of figure 10A = *R* **_A_** = 0.756), while blood flow and displacement, and blood flow and CSF flow both have moderate correlations (*R* **_B_** = 0.603; *R* **_C_** = 0.575). During exercise, the overall correlations were weaker. CSF flow and displacement had a weak correlation (*R* **_D_** = 0.363), likely due to the anticorrelated CSF flow patterns observed in exercise, particularly in the second half of the cardiac cycle. Blood flow and displacement are moderately correlated (*R* **_E_** = 0.472), whilst blood flow and CSF flow are unlikely to be correlated (*R* **_F_** = 0.228).

**Figure 11.**
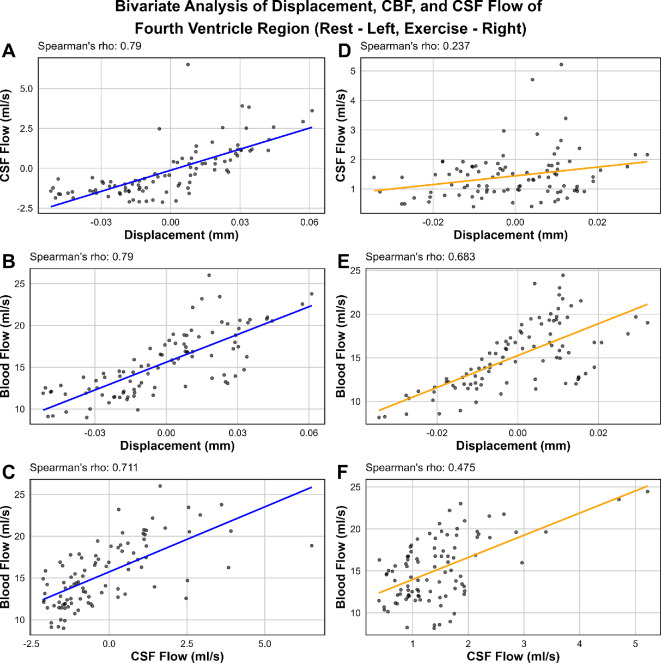
Scatter plots and bivariate correlation analyses using Spearman’s rho of displacement of the second ROI: the fourth ventricle, CBF of the internal carotid arteries, and CSF flow of C2 of the spine in rest (A–C) and exercise (D–F). Note that the data points from all five participants that met q-aMRI criteria for displacement were incorporated into these tests.

Figure 11 portrays the bivariate analyses performed on the second ROI. During rest, CSF flow and displacement, blood flow and displacement and blood flow and CSF flow are all strongly correlated (*R* **_A_** = 0.79; *R* **_B_** = 0.79; *R* **_C_** = 0.711). In contrast, during exercise, the correlations are much weaker. CSF flow and displacement are marginally correlated if at all (*R* **_D_** = 0.237), again likely due to the flow pattern changes to CSF flow during exercise. Blood flow and displacement, and blood flow and CSF flow are both moderately correlated (*R* **_E_** = 0.683; *R* **_F_** = 0.475).

## 4. Discussion

Our findings of no significant net change in blood inflow (ml s^−1^) to the cranial compartments between rest and exercise for the basilar artery and ICAs align with expectations for low-intensity, resistive exercise. This was interesting—although not unexpected—as previous studies found minimal, conflicting differences in CBF under similar conditions [15,21]. In contrast, high-intensity aerobic exercise would likely yield a significant change in CBF.

Similarly, there was no difference in the AUC measurement for both the basilar artery and ICA during exercise, indicating that cumulative flow also remained unchanged. However, when calculating the PI, both the basilar artery and ICA saw significant differences, with a decrease in pulsatility in exercise. This is expected as diastolic flow is shorter in time with the increase of heart rate, leading to a smaller difference between the maximum (peak systolic) and minimum (end-diastolic) blood flow in the blood compartment. This potentially reflects the brain’s need for consistent and elevated perfusion during elevated metabolic demand. Such a reduction in pulsatility may serve a protective function, ensuring consistent blood delivery to the brain. It was also found that heart rate increased during exercise. Since CBF remained unchanged, this suggests a reduction in stroke volume per cardiac cycle. This adjustment indicates effective cerebral autoregulation, consistent with previous literature [15], where stable blood flow is maintained without increased flow peaks to counterbalance the elevated heart rate.

A major limitation of this study regarding CBF analyses is that we did not investigate venous outflow. Although previous literature is in accordance with the Monro–Kellie doctrine finding that venous outflow increases with arterial inflow in similar exercise conditions [15], it would have been beneficial to characterize these dynamics in our acute, low-intensity exercise condition, especially considering we did not find significant changes in mean inflow.

In the aqueduct, distinct differences in CSF flow patterns were observed between rest and exercise. At rest, the flow was more oscillatory with pronounced peaks and significant regurgitation (negative flow). Exercise reduced regurgitation, producing two peaks per cycle and reflected a more consistent forward flow (from the fourth ventricle to the third ventricle via the aqueduct). This effect was confirmed by the AUC measurements, showing higher values during exercise, indicating greater cumulative net forward flow from the third to the fourth ventricle.

Similarly, in the C2 spine, rest was characterized by a more fluctuating flow, with a large dip into negative values, suggesting notable flow reversal. Exercise brought a consistent positive flow, with reduced fluctuation, indicating altered CSF dynamics. As shown in the AUC plots, the rest saw an overall cumulative net negative flow, meaning there was more regurgitation than flow down the spinal cord. Exercise resulted in a shift to a much greater, positive AUC measurement, supporting the observation of greater cumulative forward flow.

Together, these findings suggest that exercise alters CSF flow dynamics. At rest, the net flow is almost negligible. Meanwhile, exercise promoted significant net forward flow in both the aqueduct and C2, implicating CSF moving towards the spine, and leaving the intracranial space. Furthermore, we see adapting flow patterns in the aqueduct and C2 under exercise. This may be a result of the faster timeframe and/or altered brain parenchymal motion, though a future study would need to confirm this. Such changes may contribute to maintaining cerebral homeostasis and support higher CSF clearance, though further research is needed to explore the physiological implications and potential long-term effects on brain health. Further research should also assess the effect of positioning on CSF flow in these two conditions, as studies investigating CSF changes at rest using MRI found major increases in CSF flow when supine compared to the upright position [36]. Additionally, CSF production and drainage pathways should also be investigated during exercise.

Although there was no significant difference for whole brain median motion in the superior to the inferior direction (*p* = 0.063), where the largest brain motion is typically observed, each participant saw a decrease in whole brain motion post-exercise. Group difference was not statistically significant due to both the size of the sample, and the variation between participants. A larger sample of quality q-aMRI data is needed in future to confirm this trend.

Motion analysis of the third and fourth ventricles revealed a reduced amplitude of displacement during exercise compared to rest, consistent with trends seen in both CBF and CSF dynamics. The decrease in brain pulsatility, captured with q-aMRI, indicates that exercise initiates a change that decreases brain motion. Notably, q-aMRI’s capability to detect subtle subvoxel displacements enables us to quantify this physiological adaptation with a high degree of sensitivity.

Bivariate analyses with Spearman’s rho showed that intracranial dynamics are strongly correlated during rest, with the strongest correlation being CSF flow and displacement. This is apparent as the patterns of flow and displacement appear to strongly mimic each other. This finding supports the hypothesis that ventricular displacement dynamics are directly related to shifts in CSF flow. Both these metrics appear to also be strongly correlated to blood flow during rest. However, this is not the case during exercise, with only weak to moderate correlations being observed between the third ventricle, basilar CBF and aqueduct CSF flow. Correlations are slightly stronger during exercise for the second ROI (fourth ventricle, ICA CBF and C2 spinal CSF flow), but still not as strong as during rest.

Based on the correlation analyses, along with comparing intracranial dynamics qualitatively for each participant, it is seen that q-aMRI has a capacity to capture both the amplitude and frequency of displacement events, offering a deeper understanding of flow and pressure interactions within confined ventricular spaces. Furthermore, q-aMRI has the potential to estimate CSF flow pattern changes, in a 2 min three-dimensional sequence—where the current gold standard CSF flow measurements must be made in two-dimensional slices at particular ROI, and require a trained MRI technician to process these measurements. However, while this works for the entire cardiac cycle in rest, there appears to be an anticorrelation effect occurring during the second half of the cardiac cycle during exercise regarding CSF flow. Further statistical tests which are better suited to timeseries data (which may account for lag, peak and trough locations etc.) will be required in a further study to confirm these associations.

The observed correlations between CBF, CSF flow and ventricular displacement patterns have significant implications for understanding the biomechanical response of the brain to altered flow dynamics. This association, particularly evident in rest, suggests that using the combination of three-dimensional cine MRI and specialized motion estimation could serve as a valuable tool in assessing how the brain adapts to varying levels of flow changes and potentially pressure changes. The technology’s ability to quantify subvoxel motion holds promise for clinical applications, particularly in conditions with compromised CSF dynamics, such as hydrocephalus or Chiari malformations, and possibly pressure-related disorders such as idiopathic intracranial hypertension (IIH).

In pathological conditions where CSF flow and ventricular compliance are altered, estimating brain tissue displacement could offer non-invasive monitoring. For instance, by examining deviations in the flow–displacement relationship, it may be possible to detect early signs of abnormal CSF dynamics or track changes in response to treatment interventions. This technique could also help to characterize brain motion in the development of diagnostic toolkits based on specific motion patterns that may be correlated with pathophysiological mechanisms.

In this study, a pulse oximeter was used to monitor heart rate, which remained constant during the experimental session. Therefore, fatigue over the 10 min was unlikely. However, blood pressure was not monitored during the scan (only before and after), so pressure fluctuation data were not captured. Nonetheless, since we did not assign conditions based on heart rate alone and instead focused on relative differences, this limitation is unlikely to have altered our conclusions. For studies specifically investigating how intracranial dynamics vary with heart rate, the inclusion of blood pressure monitoring would provide a more comprehensive understanding of cerebrovascular responses.

At this stage, we can only apply these findings to the low-intensity, non-aerobic exercise used in this study. However, future studies involving different types of exercise will confirm the generalizability of these findings. Additionally, further investigation into the precise biomechanical pathways linking flow and displacement is warranted. Expanding the applications to examine how disease states affect these relationships could not only refine our understanding of the brain’s biomechanical responses but also contribute to the development of new diagnostic tools or targeted therapies. By leveraging the ability to quantify displacement profiles across the ventricles, future research can explore how these profiles are altered in diseases and disorders that alter brain tissue and flow pulsatility.

## 5. Conclusion

Our study shows that low-intensity exercise maintains stable CBF while promoting less pulsatile, more sustained flow patterns, inferring effective cerebral autoregulation in a healthy cohort. Exercise-induced changes in CSF dynamics suggest an adaptive mechanism supporting steady forward flow—potentially assisting with cerebral homeostasis and CSF clearance. q-aMRI and MRI-based flow analyses reveal a close correlation between ventricular displacement and CSF flow, with reduced pulsatility in the third and fourth ventricles during exercise. This highlights q-aMRI’s utility in assessing subtle brain motion changes and may offer insights for conditions with compromised CSF dynamics such as hydrocephalous, Chiari malformations and IIH. Future studies should further investigate these dynamics for various conditions for diagnostic and therapeutic applications.

## Data Availability

Concerning scan data, in line with the approved ethics documentation from this study, endorsed by the New Zealand Health and Disability Ethics Committees (NZ HDEC), and in accordance with our Indigenous and community engagement policies, non-identifiable data are available upon request and subsequent approval by the Mātai Ngā Māngai Māori Board (contacted at nmm@matai.org.nz). This protocol ensures adherence to ethical standards, respects community involvement, and upholds data sovereignty principles. Any processing software has been described in the paper, and the methods used have references to their original papers. The CBF processing software is available from Zenodo [37].
